# Bioelectrical impedance phase angle and the mortality in critically ill children

**DOI:** 10.3389/fnut.2024.1359814

**Published:** 2024-08-19

**Authors:** Jiongxian Yang, Jie Zhang, Jun Liu, Gang Liu, Suyun Qian

**Affiliations:** ^1^Department of Health Care Center, Beijing Children’s Hospital, Capital Medical University, National Center for Children’s Health, Beijing, China; ^2^Department of Pediatric Intensive Care Unit, Beijing Children’s Hospital, Capital Medical University, National Center for Children’s Health, Beijing, China

**Keywords:** electrical impedance analysis, phase angle (PhA), pediatric intensive care unit (PICU), mortality, nutrition assessment

## Abstract

**Background:**

Phase angle (PhA) is a variable obtained from bioelectrical impedance analysis (BIA). It is highly sensitive and specific and is commonly used in clinical nutrition assessment. Recently, PhA has shown promise in predicting clinical outcomes, especially as a new indicator of mortality, but its use in pediatric research is limited. This study aims to investigate the association between PhA measured at admission using BIA and PICU length of stay (LOS) and 60-day mortality in critically ill children and adolescents.

**Methods:**

A consecutive series of pediatric patients in the PICU underwent BIA measurements within 72 h of admission. All patients met the inclusion and exclusion criteria. Patient demographics, anthropometric measurements, pediatric index of mortality 2 score (PIM-2), and laboratory exams were recorded. Kaplan–Meier (K–M) survival curves were constructed based on the critical PhA value to assess differences in survival status within the 60-day window. Multivariate cox regression model was employed to illustrate the relationship between PhA and 60-day mortality rates. The Youden’s index method was used to identify the critical cut-off value for PhA in relation to mortality rates. ROC curves provided the area under the curve (AUC) and a 95% confidence interval (CI).

**Results:**

A total of 205 pediatric patients (118 boys) were included, with a mean age of 9.2 years (±6.0). Survival curves indicated a cutoff value of 3.1°, with higher survival in patients with PhA ≥3.1° compared to those with PhA <3.1° (*F* = 10.51, *p* < 0.0001). The area under the ROC curve was 0.70, with a sensitivity of 0.65 and specificity of 0.72. Total hospital LOS was longer in the PhA <3.1° group compared to the PhA ≥3.1° group (*p* = 0.000). The PhA <3.1° group had a longer PICU LOS (adjusted for age and sex, HR 1.871, *p* = 0.000, log-rank test, *p* = 0.000). PhA and PIM-2 were two independently significant correlated variables (*p* < 0.05) for the 60-day mortality rate in this study.

**Conclusion:**

Low PhA in patients is associated with longer PICU LOS and an increased risk of PICU patient mortality. PhA not only serves as an indicator for monitoring pediatric nutrition but also as a prognostic indicator for PICU patients.

## Introduction

Bioelectrical impedance analysis (BIA) is a popular technique for the assessment of body composition (1, 2), as it is non-invasive, inexpensive, quick, and utilizes portable equipment. The BIA technique is generally considered to provide clinically acceptable predictions of body composition (3). Hence, BIA has been widely used for assessing human body composition, monitoring and evaluating nutritional status and the effectiveness of nutritional therapy (1–5).

In clinical practice, BIA is commonly employed to measure electric capacitance and resistance, from which the phase angle (PhA) is calculated. PhA represents the relationship between capacitance and resistance in an angular form and provides insights into cell membrane integrity and the body’s hydration status. PhA at the frequency of 50 kHz is widely used and served as an independent nutritional indicator with high sensitivity and specificity (3, 6, 7). Studies both in adults and children have shown a strong connection between PhA and the length of hospital stay and intensive care unit (ICU) stay in various diseases (8–10), indicating its potential in predicting clinical outcomes (11–15). Several studies (9, 11) also highlighted a clear relationship between hydration obtained from BIA and clinical outcome. Additionally, Zamberlan et al. (10) proposed that PhA, as an independent indicator, was closely related to the mortality and showed a strong negative correlation with stay in pediatric intensive care unit (PICU). Results from Roche et al. (14) applied the basic BIA vector capacitance, cell resistance, and PhA to the clinical application and analysis, yielding the expected predicting outcomes.

While BIA has become increasingly popular in pediatric clinical applications, there was little research on the association between PhA and the prognosis of critically ill children in PICU. Existing research on PhA and pediatric critical illness primarily have originated from western countries, there may be differences in pediatric baseline nutritional status and ethnicity. Therefore, investigating relevant data from Chinese children is of significant practical importance. This single-center study aims to analyze the correlation between whole-body PhA at 50 kHz obtained from multiple-frequency BIA (MFBIA) and PICU LOS and mortality, aiming to provide critical care physicians with insights into the clinical utility of PhA beyond nutritional assessment.

## Materials and methods

### Study design and population

It was a prospective observational single-center study. We consulted professional statistical experts to help with design, sample size calculation and statistical analysis. The sample size was determined from a power analysis. Specifically, the sample size was calculated assuming an HR of 1.5 for PhA in the 60-day mortality of critically ill children, with a 60-day mortality rate of approximately 50%, *α* = 0.05, and *β* = 0.1. We finally completed the data collection within two natural years, which not only satisfied the sample size, but also avoided the influence of seasonal factors on the outcome.

Patients admitted to PICU of Beijing Children’s Hospital were collected continuously, from October 2019 to October 2021. We finally included 205 subjects.

Inclusion criteria were as follows: age between 3 and 18 years, intact skin on extremities without severe wounds or bandages, no implanted metallic objects, and a PICU length of stay (LOS) exceeding 72 h.

Exclusion criteria included traumatic injuries (e.g., accidents or falls), continuous use of extracorporeal circulation, hemodynamic instability, inability to complete measurements due to other reasons, and a PICU LOS less than 72 h (discharged or clinical death).

### Methods

BIA measurements were conducted within 72 h of admission to the PICU. Data collected included sex, age, laboratory test results, pediatric index mortality score 2 (PIM-2), anthropometric measurements, nutritional screening, BIA data within 72 h, PICU LOS, total hospital LOS, and mortality status.

### Anthropometric measurements

#### Height

For ambulatory patients, height (H) was measured using a stadiometer. For bedridden patients, parental reports were used, and supine length measurements were taken using a measuring tape, accurate to 0.5 cm.

#### Weight

Weight (Wt) was measured using the hospital beds, which had integrated weight measurement functionality.

#### Body mass index

Body mass index (BMI) was calculated as BMI = Wt (kg)/H^2^ (m^2^). Nutritional status was determined using BMI-for-age *Z*-scores based on reference values from the World Health Organization (WHO). *Z*-scores were calculated using WHO Anthro (0–5 years) and WHO Anthro (5 years and older) to classify patients as follows: severe underweight (BMI *Z*-score < −3), underweight (BMI *Z*-score < −2), normal (−2 ≤ BMI *Z*-score ≤ +1), overweight (BMI *Z*-score > +1), obesity (BMI *Z*-score > +2).

### Bioelectrical impedance analysis

A special type of MFBIA machine (Biospace In-Body S10, Seoul, Republic of Korea) was used, and all the measurements obtained from one machine. The MFBIA analysis involved six-frequency (1, 5, 50, 250, 500, 1,000 kHz) and eight-electrode (thumb, middle finger, medial ankle, lateral ankle), and was performed in five segments trunk, left upper limb, right upper limb, left lower limb and right lower limb. It can provide relatively accurate estimations of body composition for the pediatric study (16, 17).

MFBIA measurements were performed within 72 h of admission, following strict operation instructions and performed in the supine position. Patients were positioned with their feet separated by 10–15 cm, and their upper limbs abducted at a 15° angle from their body sides. MFBIA measurements were obtained after a 2 h cessation of enteral or parenteral nutrition. Before measurement, patients were required to maintain the lying position for more than 15 min, and the electrodes’ contact sites and skin were cleaned with a moist tissue. Eight-point tactile-electrode were placed on the patient’s thumbs and middle fingers and two sides of the ankles. MFBIA measurements were directly displayed on the device. After measurements, the electrodes and device wires were disinfected with sterilization wipes. Various parameters including total body water (TBW), extracellular water (ECW), intracellular water (ICW), edema index (ECW/TBW), body cell mass (BCM), fat mass (FM), fat-free mass (FFM), body fat percentage (BFP), visceral fat area (VFA), and whole-body PhA at 50 kHz were obtained directly. Furthermore, lean body mass index (LBMI) was calculated LBMI = FFM (kg)/H (m)^2^.

In this article, whole-body PhA at 50 kHz was used for analysis, referenced as PhA.

### Nutrition screening

Nutrition screening was conducted using the Pediatric Yorkhill Malnutrition Score (PYMS) scale. PYMS was administered by a nutritionist within 48 h of admission. A score of ≥2 on the PYMS scale indicated a high nutrition risk.

### Laboratory tests

Laboratory tests included the measurement of total protein (TP), albumin (ALB), and prealbumin levels.

### Clinical outcome measures

A 60-day observation period was used to assess mortality rates. PICU LOS and total hospital LOS exceeding 60 days were determined within this 60-day window.

### Statistical methods

Data analysis was conducted using SPSS 22.0 software. Descriptive statistics, including sex and age, anthropometric measurements, and clinical outcomes (PICU LOS, mortality rates), were presented as mean ± standard deviation (M ± SD).

Kaplan–Meier (K–M) survival curves were constructed based on the critical PhA value to assess differences in survival status within the 60-day window. Multivariate cox regression model was employed to illustrate the relationship between PhA and 60-day mortality rates. The Youden’s index method was used to identify the critical cut-off value for PhA in relation to mortality rates. ROC curves provided the area under the curve (AUC) and a 95% confidence interval (CI).

### Ethics

This study received approval from the Ethics Committee of Beijing Children’s Hospital, Capital Medical University (2023-E-058-R).

## Results

### General characteristics

#### Demographic information

This prospective observational study included a total of 205 pediatric patients, of whom 118 were boys (52.7%). All patients met the inclusion criteria and completed the assessments. The average age was (9.2 ± 6.0) years, with an average PIM-2 score of (10.4 ± 14.2), and an average PhA of (3.6 ± 1.4)°. The mean total hospital LOS was (34.5 ± 18.7) days, and the average PICU LOS was (25.6 ± 18.6) days. A ROC curve revealed a critical cut-off value for PhA at 3.1° (see the details of the ROC curve later). Patients were then divided into two groups based on PhA 3.1° and their characteristics were described. There were no statistically significant variations in sex, age, albumin, BMI-*Z* score, PYMS score, body fat percentage, lean body mass index and prealbumin levels between the two groups. However, significant differences were noted in PhA, PIM-2 score, total hospital LOS and PICU LOS. Additional details can be found in Table 1.

**Table 1:** Demographic information and basic data.

Parameters	PhA ≥3.1°	PhA <3.1°	Total
Number (cases)	125 (65 males)	80 (43 males)	205 (108males)
Age (years)	8.9 ± 4.9	9.3 ± 6.2	9.2 ± 6.0
Total hospital LOS^*^ (ds)	31.0 ± 18.2	38.5 ± 18.5	34.5 ± 18.7
PICU LOS^*^ (ds)	20.8 ± 15.3	31.2 ± 20.6	25.6 ± 18.6
PIM-2 score^*^	8.7 ± 12.9	13.7 ± 17.6	10.4 ± 14.2
Albumin (g/L)	34.5 ± 6.6	31.6 ± 5.2	33.1 ± 5.8
BMI *Z*-score	−0.4 ± 3.1	0.2 ± 2.9	−0.6 ± 2.6
PYMS score	3.2 ± 1.6	3.4 ± 2.1	3.4 ± 1.8
PhA (°)^*^	4.3 ± 1.0	2.3 ± 0.4	3.6 ± 1.4
Body fat (%)	19.2 ± 14.6	17.6 ± 13.8	18.7 ± 13.9
LBMI	13.8 ± 6.8	14.9 ± 4.2	14.2 ± 5.6
Prealbumin (mg/L)	140. ± 67.2	129.0 ± 58.4	138.8 ± 63.0

^*^Represent *p*-values <0.05; LOS, length of stay; PIM-2, pediatric index of mortality 2 score; Alb, albumin; PYMS, Pediatric Yorkhill Malnutrition Score; PhA, phase angle (whole body phase angle at 50 kHz); LBMI, lean body mass index.

#### BMI distribution

Nutritional status was assessed based on BMI *Z*-scores, with 58 patients (28.5%) classified as underweight or malnourished, 66 patients (32.2%) as overweight or obese, and 81 patients (39.2%) as having a normal nutritional status. The PhA values for each group are provided in Table 2. The mortality rates for the underweight/malnourished group, normal group, and overweight/obese group were 15.8, 11.4, and 11.1%, respectively. There was no statistically significant difference in mortality rates between the groups (*F* = 0.828, *p* = 0.439), and PhA values also showed no statistically significant differences between the groups (*F* = 0.475, *p* = 0.623).

**Table 2:** BMI categories and corresponding PhA levels.

Nutritional status	Cases (*n*) (%)	*Z*_BMI/age_	PhA (°)
Underweight and severe underweight (*Z* < −2)	58 (28.5%)	−4.7 ± 3.6	2.9 ± 1.6
Normal (−2 ≤ *Z* ≤ 1)	81 (39.3%)	−0.2 ± 0.8	3.3 ± 1.2
Overweight and obesity (*Z* >1)	66 (32.2%)	1.7 ± 0.6	3.5 ± 1.2

BMI *Z*-score < −3 indicates severe underweight, *Z*-score < −2 indicates underweight, −2 ≤ *Z*-score ≤ +1 indicates normal, *Z*-score > +1 indicates overweight, *Z*-score > +2 indicates obesity.

#### Distribution of diseases

Among the 205 pediatric patients in the study, 55 (26.8%) were surgical patients, while 150 had internal medical conditions. The primary medical conditions included severe infections in 96 cases (including 29 cases of septic shock), blood system tumors in 22 cases, and other conditions in 32 cases. There were 25 deaths, with 5 cases from surgical diseases and 20 cases from internal medical conditions. Total hospital LOS, PICU LOS, PIM-2, and PhA all exhibited statistically significant differences between the deceased and surviving patient groups. Please refer to Table 3 for detailed information.

**Table 3:** Comparison of characteristics between surviving and deceased patients.

Parameters	Survivors (180 cases)	Non-survivors (25 cases)	*T*	*p*
Age (years)	9.3 ± 6.2	8.1 ± 3.9	−0.992	0.322
Total hospital LOS (ds)	36.0 ± 18.6	19.3 ± 12.9	13.035	0.000
PICU LOS (ds)	26.8 ± 19.1	12.7 ± 5.1	10.996	0.000
PIM-2 score	8.8 ± 10.12	25.0 ± 27.9	−16.153	0.000
Alb (g/L)	33.3 ± 6.0	31.7 ± 3.9	0.404	0.686
BMI *Z*-score	−1.2 ± 3.6	−0.8 ± 2.6	−1.872	0.061
BFP (%)	18.4 ± 14.1	20.9 ± 12.0	−0.846	0.398
LBMI	14.19 ± 3.98	14.81 ± 12.91	−0.241	0.810
PhA (°)	3.6 ± 2.4	2.9 ± 0.7	4.313	0.000
Prealbumin (mg/L)	137.9 ± 62.8	148.5 ± 65.5	−0.742	0.459

LOS, length of stay; PIM-2, pediatric index of mortality 2 score; Alb, albumin; BMI, body mass index; BFP, body fat percentage; LBMI, lean body mass index; PhA, phase angle (whole body phase angle at 50 kHz).

### ROC curve for PhA and mortality in critically ill children

**Figure 1:**
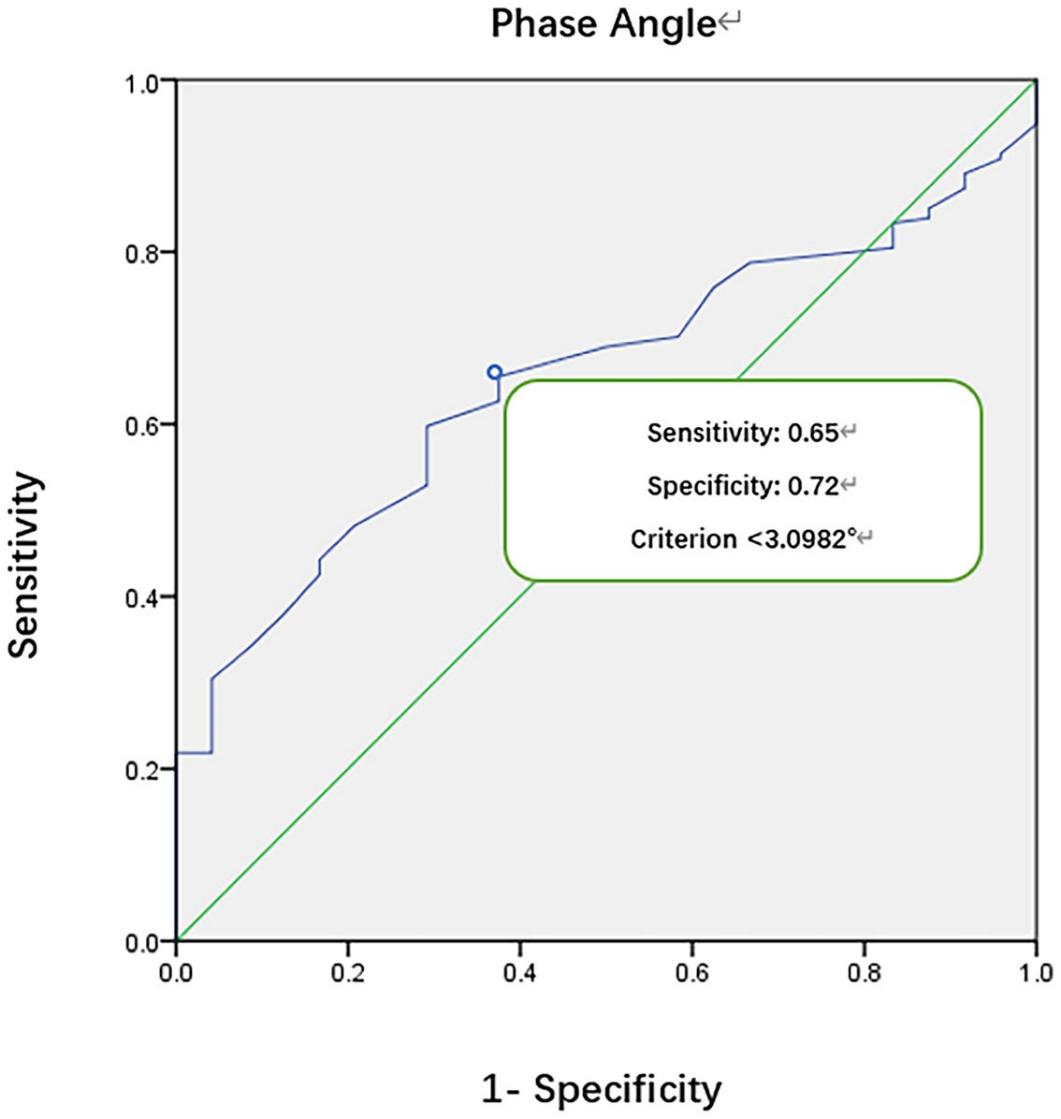
ROC curve of phase angle vs. mortality in children and adolescents in the PICU.

A ROC curve was generated to assess the relationship between PhA and mortality in critically ill children, revealing a critical cut-off value for PhA at 3.1° (Figure 1). The area under the ROC curve was 0.70, with a 95% confidence interval of (0.62, 0.78). The sensitivity was 0.65, and the specificity was 0.72.

### PhA and survival curve, LOS, and Cox regression model in critically ill children

**Figure 2:**
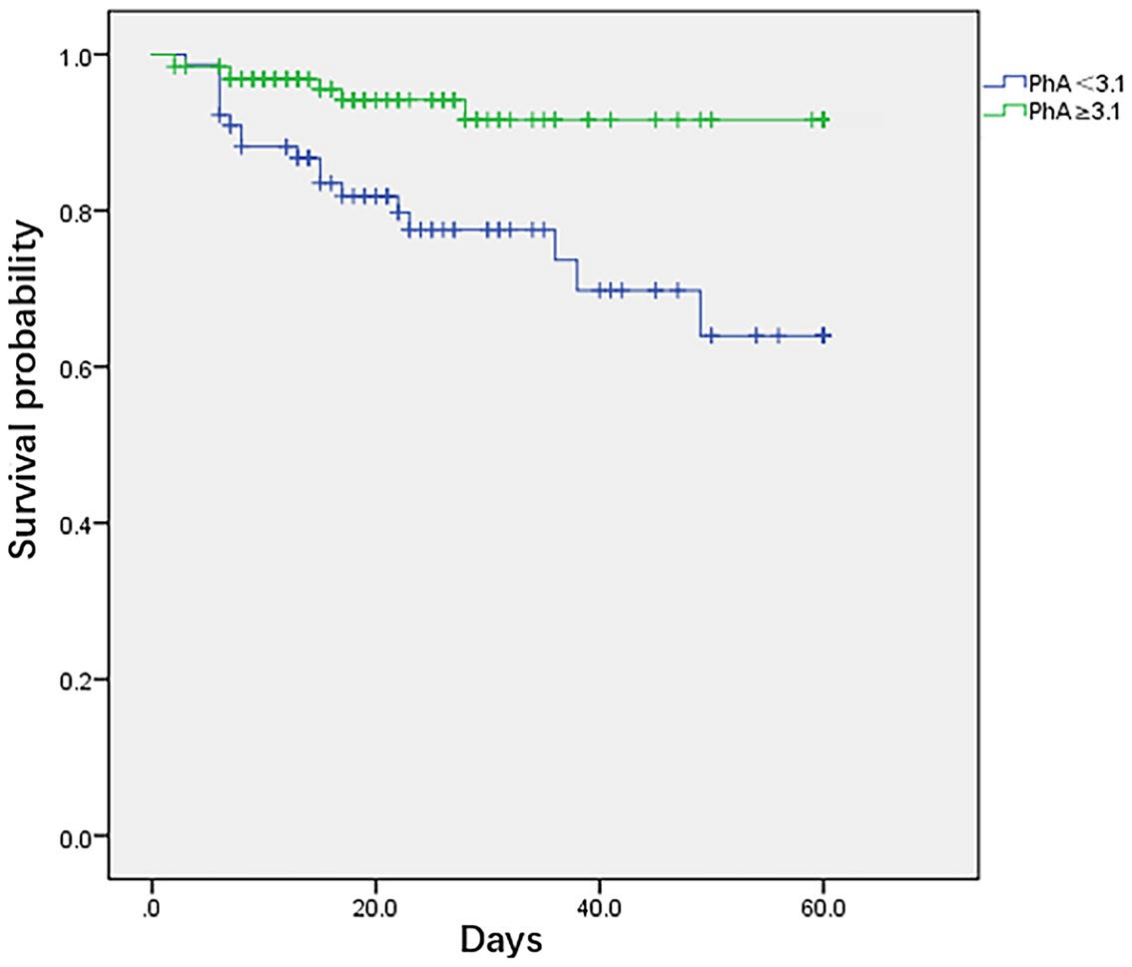
Kaplan–Meier survival curves for critically ill children and adolescents using cut-off based on phase angle obtained by bioelectrical impedance analysis.

Using the critical cut-off value obtained from the ROC survival curve, it was observed that the survival rate of the PhA ≥3.1° group was significantly higher than that of the PhA <3.1° group (*F* = 10.51, *p* = 0.001) (Figure 2). The PICU LOS for the PhA ≥3.1° group and PhA <3.1° group was (20.8 ± 15.3) days and (31.2 ± 20.6) days, respectively (*T* = −14.854, *p* = 0.000). After adjusting for age and sex in the Cox regression analysis, it was found that children with PhA <3.1° had a longer PICU LOS [HR 1.871, 95% CI (1.355, 2.584), *p* = 0.000]. The total hospital LOS for the PhA ≥3.1° group and PhA <3.1° group was (31.0 ± 18.2) days and (38.5 ± 18.5) days, respectively (*T* = −10.639, *p* = 0.000).

Initially, our Cox regression models included age, sex, total hospital LOS, LOS in PICU, PIM-2 score, albumin, BMI *Z*-score, PYMS score, PhA, body fat, LBMI, and prealbumin. We calculated the variance inflation factor (VIF) for the Cox regression models. If the VIF was greater than 10, we considered that the two variables exhibited multicollinearity. We calculated the variance inflation factor (VIF) for the Cox regression models. If the VIF was greater than 10, we considered that the two variables exhibited multicollinearity. Based on Cox regression modeling and multicollinearity analysis, both PIM-2 score and PhA were identified as two independently significant correlated variables for the 60-day mortality rate in this study (*p* < 0.05) (Table 4).

**Table 4:** Mortality estimates for critically ill children and adolescents from a Cox regression model (PhA).

Variable	HR	95% CI	*p*
Age	0.997	0.855–1.162	0.965
Sex	0.903	0.34–2.394	0.837
BMI *Z*-score	0.927	0.864–1.093	0.635
LBMI	0.986	0.933–1.041	0.606
PIM-2	1.030	1.008–1.052	0.006
PhA	0.542	0.299–0.983	0.044
Alb	1.005	0.907–1.115	0.919
Prealbumin	0.992	0.981–1.002	0.115

BMI, body mass index; LBMI, lean body mass index; PIM-2, pediatric index of mortality 2 score; PhA, phase angle (whole body phase angle at 50 kHz); Alb, albumin.

## Discussion

Critically ill children often present with complex medical conditions, and there is a clinical demand for convenient, non-invasive, accurate, and stable prognostic indicators. PhA has been increasingly recognized as a valuable clinical assessment parameter (18). This study highlights a strong association between PhA as an independent factor and the prognosis of pediatric patients in the PICU, showing good sensitivity and specificity. In contrast, clinical nutritional indicators such as BMI, ALB, prealbumin, and LBMI did not exhibit predictive value for the prognosis of critically ill children.

A low PhA indicates either cell death or reduced cellular performance (19). It serves as an independent risk factor for mortality in critically ill children, demonstrating heightened sensitivity and specificity. In the results of this study, ROC curve analysis suggests a PhA threshold of 3.1° for the study population, with a sensitivity of 0.65 and specificity of 0.72. The mortality rate in the low PhA group (<3.1°) is significantly higher than in the high PhA group (≥3.1°), leading to a notable difference in survival curves between the two groups (*p* = 0.0001). According to Cox regression analysis, the HR for PhA in relation to mortality in critically ill children is 0.542 (*p* = 0.044). Children in the low PhA group experience longer PICU LOS, with an adjusted HR of 1.871 after correcting for age and sex (*p* = 0.000). Zamberlan et al. (10) similarly applied BIA to critically ill children aged 2 months to 18 years. They established a PhA threshold of 2.8°, with significantly increased mortality rates in critically ill children with PhA ≤2.8° compared to those with PhA >2.8°. Cox regression analysis also indicated PhA and PIM-2 scores as two independent risk factors for PICU children. The PhA threshold of 3.1° in our study closely approximates the 2.8° threshold from their results. Sepsis remains one of the most common conditions in the PICU. Azevedo et al. (20) conducted BIA testing on pediatric sepsis patients to explore whether bioelectrical impedance parameters could serve as predictors of septic shock. Their results indicate a higher incidence of septic shock and longer ICU LOS with lower reactance normalized by height (Xc/H) and PhA medians (Xc/H: *p* < 0.0001, PhA <0.0006). However, these parameters have limitations when used independently for predicting septic shock. For instance, when Xc/H falls below 48.63 ohm/m upon admission, the incidence of septic shock significantly increases during hospitalization, but the exact timing of onset remains unpredictable. Lower PhA values correlate with higher odds ratios (OR) but lack a similarly defined threshold. Nonetheless, this study recommends monitoring both these parameters in critically ill children.

PhA is known to be closely associated with factors such as age, sex, and nutritional status. Zamberlan’s et al. (10) study identified a PhA threshold of 2.8° associated with mortality, while in contrast, this study establishes a threshold of 3.1°. This difference may be attributed to variations in the study populations, types of diseases, and age distributions. The observed population in this study comprises children aged 3 to 18 years with both internal medical conditions and various non-traumatic surgical conditions, excluding trauma and accidents. Conversely, Zamberlan’s et al. (10) study group focused on pediatric patients with chronic internal medical conditions aged 2 months to 18 years.

The dynamic changes in PhA can provide better insights into disease progression. An increase or decrease in PhA indicates changes in cellular vitality and integrity, such as alterations in cell size, membrane permeability, and cellular components, which are related to various clinical outcomes. Coradine et al. (21) investigated the clinical prognosis of PhA in neonates and preterm infants. They conducted initial screening within 24 h of admission and repeated tests at 24 h, 48 h, and 7 days. The results revealed that a declining PhA within 48 h of admission was a predictive indicator of neonatal mortality. While studies in critically ill children and neonates consistently highlight the significant correlation between low PhA and mortality, our study did not dynamically monitor changes in PhA. Further research could explore the relationship between dynamic changes in PhA and prognosis.

Numerous pediatric nutritional assessment indicators are commonly employed in clinical practice, with LBMI proving to be a more effective measure of lean body weight and muscle reserve, closely linked to prognosis (22). In this study, various frequently used indicators, including BMI, ALB, prealbumin, and LBMI, were individually examined to investigate their associations with clinical outcomes. Based on a 60-day clinical endpoint observation, patients were categorized into survival and non-survival groups. No statistically significant differences were found between these groups in terms of BMI, ALB, prealbumin, or LBMI. Furthermore, Cox regression analysis revealed no significant correlations between BMI, ALB, prealbumin, or LBMI and prognosis. PICU patients often present severe illness, rapid clinical changes, and multiple clinical complications, which may limit the utility of these commonly used indicators in predicting the prognosis of critically ill children.

This study did not stratify children with different diseases for further investigation and did not dynamically monitor changes in PhA among the study participants and their relationship with prognosis. The relationship between other parameters such as TBW (ICW and ECW), BCM and VFA and prognosis was not analyzed, so their predictive value in the prognosis of PICU patients remains uncertain and need further research. Additionally, it is a single-center study with a limited sample size. Therefore, expanding the sample size, categorizing patients based on their underlying diseases, dynamically monitoring PhA changes, and exploring critical points in the relationship between PhA and mortality in different PICU disease groups could enhance the clinical application of PhA and guide clinical nutrition diagnosis and treatment more effectively.

## Conclusion

This study highlights that PICU patients with PhA <3.1° experience a substantially higher mortality rate compared to those with PhA ≥3.1°. PhA emerges as an independent influencing factor for mortality in PICU children. Given its non-invasive nature, convenience, and reasonable predictive ability regarding the risk of mortality in PICU patients, PhA holds promise for widespread utilization in pediatric practice.

## Data Availability

The original contributions presented in the study are included in the article/supplementary material, further inquiries can be directed to the corresponding author.
